# Adolescent Self-Organization and Adult Smoking and Drinking over Fifty Years of Follow-Up: The British 1946 Birth Cohort

**DOI:** 10.1371/journal.pone.0146731

**Published:** 2016-01-11

**Authors:** Atsushi Nishida, Dorina Cadar, Man K. Xu, Timothy Croudace, Peter B. Jones, Diana Kuh, Marcus Richards, MRC National Survey of Health and Development scientific and data collection team

**Affiliations:** 1 Department of Psychiatry & Behavioural Science, Tokyo Metropolitan Institute of Medical Science, Tokyo, Japan; 2 MRC Unit for Lifelong Health and Ageing at UCL, London, United Kingdom; 3 Departments of Psychiatry and Public Health, University of Cambridge, Cambridge, United Kingdom; 4 Department of Health Sciences and Hull York Medical School, University of York, York, United Kingdom; 5 Department of Psychiatry, University of Cambridge, Cambridge, United Kingdom; 6 CAMEO, Cambridgeshire & Peterborough NHS Foundation Trust, Cambridge, United Kingdom; Aichi Cancer Center Research Institute, JAPAN

## Abstract

Variations in markers of adolescent self-organization predict a range of economic and health-related outcomes in general population studies. Using a population-based birth cohort study we investigated associations between adolescent self-organization and two common factors over adulthood influencing health, smoking and alcohol consumption. The MRC National Survey of Health and Development (the British 1946 birth cohort) was used to test associations between a dimensional measure of adolescent self-organization derived from teacher ratings, and summary longitudinal measures of smoking and alcohol consumption over the ensuing five decades. Multinomial regression models were adjusted for sex, adolescent emotional and conduct problems, occupational social class of origin, childhood cognition, educational attainment and adult occupational social class. With all covariates adjusted, higher adolescent self-organization was associated with fewer smoking pack years, although not with quitting; there was no association with alcohol consumption across adulthood (none or heavy compared with light to moderate). Adolescent self-organization appears to be protective against smoking, but not against heavy alcohol consumption. Interpretation of this differential effect should be embedded in an understanding of the social and sociodemographic context in which these health behaviours occur over time.

## Introduction

Self-organization has been defined as “effortful regulation of the self by the self” [1], and has various synonyms and related concepts, including self-regulation, self-control, conscientiousness [1], self-efficacy and mastery [2]. Self-organization in childhood is associated with a wide range of outcomes in the general population. In the New Zealand Dunedin study this predicted socioeconomic attainment and risk of single-parent child-rearing, poor physical health, substance dependence, and criminal conviction [3]. By way of explanation, these authors refer to the “snares” that trap such adolescents into harmful lifestyles; for example, Dunedin participants with low self-organization were likely to begin smoking by age 15 years [3]. Since smoking is a major cause of premature mortality in high income countries [4], and since it tends to cluster with other risky health behaviours, including heavy alcohol intake [5], these health behaviours are likely to be an important mediator of the poor health outcomes observed in the Dunedin study.

The Medical Research Council National Survey of Health and Development (NSHD), also known as the British 1946 birth cohort, offers an excellent opportunity for a detailed investigation of smoking and alcohol consumption in relation to self-organization, since the latter was derived from independent teacher ratings when study members were in early adolescence, and repeated measures of smoking and alcohol consumption were obtained over the life course, along with a wide range of potential confounders and mediators, including adolescent emotional and conduct problems. We hypothesised that adolescent self-organization is independently and inversely associated with smoking and potentially hazardous levels of alcohol consumption.

## Materials and Methods

### Study population

The NSHD originally consisted of a socially stratified sample of 5362 singleton children (girls: n = 2547; boys: n = 2815) born within marriage in one week in March 1946 in mainland Britain, with regular follow-up across life [6], most recently between 2006 and 2010, at 60–64 years. At the latter wave 3136 study members were sent a postal questionnaire. Contact was not attempted with those who were already known to have died (n = 718), who were living abroad (n = 567), had previously withdrawn from the study (n = 594) or were permanently untraced (n = 320) [7]. Of the 2462 who returned a postal questionnaire, 2856 with a known address in England, Scotland or Wales were invited for an assessment at one of six clinical research facilities (CRFs) or to be visited by a research nurse at home. Of these, 1690 completed a clinic visit and 539 completed a home visit. The current study protocol received ethical approval from the Greater Manchester Local Research Ethics Committee for the four English sites and from the Scotland A Research Ethics Committee. Written informed consent was obtained from the study member at each stage of data collection.

### Adolescent mental health

Teachers were asked to rate study members on a three category response scale comparing their behaviour to that of “a normal child” for the first time at age 13, and then again at 15 years using items that were forerunners of those used in the Rutter A scale [8]. Previous work used factor analysis of these ratings to identify two dimensions, emotional and conduct problems [9,10]. Subsequently, teacher rating data at age 13 and 15 years were re-subjected to separate categorical data exploratory factor analysis at these ages, suggesting a three factor solution for these items, representing emotional problems (e.g. gloomy and sad, extremely fearful), conduct problems (e.g. disobedience, evading truth to keep out of trouble) and self-organization [11]. The self-organization factor was defined by items relating to: attitude to work; concentration; neatness in work; and not daydreaming in class [11]. Factor scores at ages 13 and 15 years were summed to create scales representing these dimensions, and were standardized to a mean of 0 and SD of 1.

### Smoking

Lifetime smoking was represented by pack years per person, i.e. the average number of cigarettes smoked per day divided by 20 (the number of cigarettes in a standard pack), multiplied by the number of years of smoking. In NSHD the pack-years variable was initially derived using data on smoking frequency from six interview waves, at age 20, 25, 31, 36, 43 and 53 years [12]. At each of these ages study members were asked about their smoking status (current, former, and never) and the number of cigarettes they smoked per day. At ages 20 and 25 years they were also asked about age at smoking initiation. For the present analyses pack-years was updated to the subsequent wave at 60–64 years. This was constructed by summing data from seven study periods: 20 years and under, 20–25 years, 25–31 years, 31–36 years, 36–43 years, 43–53 years and 53–60–64 years. For each period, the amount smoked at the start of the period was multiplied by half the length of the period and then summed with the amount smoked at the end of the period, which was also multiplied by half the length of the period. Study members who never smoked were assigned a pack-years value of zero. To construct overall pack-years various selection and imputations were made. First, the median age at smoking initiation (16 years) was imputed for current smokers at age 20 years with missing age at initiation. Second, study members with missing smoking data on three or more consecutive occasions were dropped from analyses. Third, if the study member was a current smoker at the beginning of the period and an ex-smoker at the end, then pack-years were calculated for half the length of the period, and the other half was set to zero. A similar procedure was applied if the study member was a nonsmoker at the start and then became a smoker at the end of the period. Third, if smoking status for a period was known but not the amount smoked, the amount was imputed from adjacent time periods. Finally, when summing pack years study members with more than one period missing were dropped, but for those with one period missing the pack-years in this period were imputed based on the mean of the other periods. For distributional reasons this continuous pack years variable was grouped into lifetime non-smokers (those with a zero value), light to moderate smokers (up to 20 pack years, equivalent to less than 10 cigarettes per day over the 45 year interval), and heavy smokers (greater than 20 pack years). A binary variable was also calculated at age 60–64 representing current smoking versus quitting. If study members were not current smokers at this age but had smoked in the past they were asked about age at quitting. The latter was then grouped into: within 1 year; 1–5 years; 5–10 years, and more than 10 years.

### Alcohol consumption

Alcohol consumption was assessed using diet diaries at 36, 43, 53 and 60–64 years, explained by a research nurse. Study members were asked to record all foods and drinks consumed, including alcoholic, over 5 consecutive days using household measures. At some waves they additionally completed a 2-day recall, but this was not used in the present study. Alcoholic content was converted to grams per 100ml, based on a study of the average alcohol content of 29 beers, ciders, wines, liqueurs and spirits, derived from samples of each type [13]. Mean daily alcohol (grams) was calculated over the five day entries. This in turn was converted to units (U) based on UK government guidelines, where one unit represents 8g of pure alcohol. Based on UK Department of Health guidelines [14], separate categories of alcohol consumption were created for men and women: 0 units/day (none/abstainers), 0–4 units/day (light or moderate drinkers) and more than 4 units/day (heavy drinkers) for men; and 0 units/day (none/abstainers), 0–3 units/day (light or moderate drinkers), and more than 3 units/day (heavy drinkers) for women. An overall measure of alcohol consumption across four study periods in adulthood was calculated as the average of daily intakes at all ages (when data was available for at least three out of four waves); for consistency with the smoking outcome, this was recoded as no consumption, light to moderate and heavy consumption across midlife.

### Covariables

The following covariables were used: sex, father’s occupational social class and childhood cognitive ability as potential confounders; educational attainment and midlife occupational social class as potential mediators [15]. Father’s occupational social class was based on the UK Registrar General’s classification when study members were aged 11 years, or if this was unknown, aged 4 or 15 years. Childhood cognitive ability at 8 years was represented as the sum of four tests of verbal and nonverbal ability [16]. Educational attainment by 26 years was dichotomized into those with at least advanced (‘A level’, taken during the final year of secondary/high school) or higher (university or equivalent) qualifications, vs. those below this level. Midlife occupational social class was used at 53 years, or earlier than this if missing, coded according to the UK Registrar General.

### Statistical analyses

F-test and chi-square tests, as appropriate, were used for initial bivariable analysis of associations between the independent variables and the smoking and alcohol outcomes. Since the latter were both coded into three levels, multivariable multinomial logistic regression analyses were then conducted to test associations between adolescent self-organization and these outcomes. This was conducted in four stages: Model 1 was adjusted for sex only; Model 2 was further adjusted for adolescent emotional and conducts problems; Model 3 was further adjusted for the potential confounders of social class of origin and childhood cognition; Model 4 was further adjusted for the potential mediators of educational attainment and adult social class. Sex x adolescent mental health variable interactions on each health behaviour were not statistically significant; therefore all analyses were adjusted rather than stratified by sex. For the smoking variable the lifetime non-smoking category was used as the reference group; for alcohol the reference category was the overall light to moderate consumption category, in view of considerable evidence that, compared to this group, a range of health outcomes are poorer with abstention and heavy consumption [17]. Finally, logistic regression was used for a sub-analysis of current smokers versus quitters, with the same covariates as above. This was initially tested as current smoking versus quitting at any time. However, since reasons for recent quitting may be different in some study members than for long-term quitting (e.g. a response to health problem rather than a health preventive choice), this was also tested as current smoking or quitting within 10 years versus quitting over 10 years ago. For all multivariable analyses, the adolescent mental health and childhood cognition variables were each standardized to a mean of 0 and SD of 1. All analyses used SPSS version 21.

## Results

Sample size with non-missing data for adolescent mental health, smoking, alcohol consumption and all covariates was 1689. Those with missing data for smoking or alcohol consumption had significantly higher levels of adolescent mental health problems, lower adolescent self-organization, lower childhood cognitive scores, were more likely to be of manual social (origin and adult) and less likely to have advanced education (p<0.05 for all comparisons, with the single exception of social class of origin in relation to missing data for smoking; p = 0.97). Consistent with this, there was a graded increase from lowest to highest tertile of adolescent self-organization in likelihood of providing data at the age 60–64 assessment, compared to non-response at this assessment due to death, emigration, refusal, inability to trace and permanent loss to follow-up (p<0.001).

Table 1 shows descriptive information and bivariable p values for associations between each adolescent mental health score and each covariate (raw scores for the adolescent mental health and childhood cognition variables), and the smoking and alcohol outcomes. Mean level of adolescent self-organization was inversely associated with smoking pack years but showed an inverted-U trend with level of alcohol consumption over adulthood. Inverse linear associations were clearly evident between emotional problems and smoking and alcohol consumption. Conduct problems were positively associated with smoking but showed a J-shaped trend with alcohol. Women were less likely to be heavy smokers or to consume alcohol. Manual occupational social class, of origin and in adulthood, was positively associated with smoking but with lower alcohol consumption. Childhood cognition and educational attainment were inversely associated with smoking but positively associated with alcohol, with these trends particularly strong for education.

**Table 1:** Descriptive information (means and SDs or proportions) and corresponding p values for bivariate between-group tests for each health behavior.

**Smoking pack years**	**Lifetime non**	**Light to moderate**^**1**^	**Heavy**^**2**^	**p**
N	575	725	389	
Self-organization	1.61 (1.38)	1.10 (1.40)	0.81 (1.28)	<0.001
Emotional problems	0.12 (1.44)	-0.09 (1.37)	-0.21 (1.23)	0.001
Conduct problems	-0.53 (1.18)	0.11 (1.37)	0.42 (1.34)	<0.001
Sex (% female)	57.6	47.7	44.5	<0.001
Social class of origin (% NM)	45.0	43.3	28.3	<0.001
Childhood cognition	92.29 (27.06)	91.85 (27.74)	83.39 (26.35)	<0.001
Education (% advanced)	45.7	37.4	18.0	<0.001
Adult social class (% NM)	75.3	66.9	48.8	<0.001
**Alcohol consumption over adulthood**	**None**	**Light to moderate**^**3**^	**Heavy**^**4**^	p
N	158	1256	275	
Self-organization	0.99 (1.28)	1.28 (1.40)	0.97 (1.43)	0.001
Emotional problems	0.54 (1.61)	-0.06 (1.33)	-0.34 (1.30)	<0.001
Conduct problems	-0.04 (1.41)	-0.10 (1.34)	0.28 (1.36)	<0.001
Sex (% female)	71.5	54.1	21.1	<0.001
Social class of origin (% NM)	29.7	42.0	39.6	0.01
Childhood cognition	78.53 (25.87)	90.87 (27.24)	92.96 (27.61)	<0.001
Education (% advanced)	17.7	36.5	42.5	<0.001
Adult social class (% NM)	49.4	68.9	60.0	<0.001

^1^≤20 pack years.

^2^>20 pack years.

^3^representing 0–4 units/day (men); 0–3 units/day (women).

^4^representing >4 units/day (men); >3 units/day (women).

Table 2 shows the multinomial odds ratios for light to moderate and heavy smoking pack years compared to the no smoking reference category. Self-organization was strongly inversely associated with smoking in the analysis adjusted for sex only (Model 1). These odds ratios were modestly attenuated by adjustment for adolescent emotional and conduct problems (Model 2). Additional adjustment for social class of origin and childhood cognition (Model 3) further attenuated the odds ratio for heavy smoking. This was also the case after final adjustment for educational attainment and adult social class (Model 4), although associations remained significant at the 5% level. Based on information provided at age 25, only 15 study members (less than 1% of the analytic sample) reported that they were regular smokers before age 15 years, when the teacher ratings relating to self-organization were first made.

**Table 2:** Multinomial logistic regression coefficients for odds of light to moderate and heavy smoking pack years (ref. = lifetime non-smoking)

	Model 1	Model 2	Model 3	Model 4
Self-organization:				
Light to moderate smoking	0.69 (0.61, 0.77) p<0.001	0.80 (0.68, 0.94) p = 0.006	0.77 (0.65, 0.91) p = 0.002	0.80 (0.67, 0.94) p = 0.008
Heavy smoking	0.56 (0.49, 0.64) p<0.001	0.65 (0.54, 0.79) p<0.001	0.71 (0.58, 0.87) p = 0.001	0.79 (0.64, 0.97) p = 0.02
Female sex:				
Light to moderate smoking	0.74 (0.59, 0.92) p = 0.008	0.79 (0.63, 0.99) p = 0.04	0.79 (0.63, 1.00) p = 0.05	0.76 (0.59, 0.96) p = 0.02
Heavy smoking	0.69 (0.52, 0.90) p = 0.006	0.76 (0.58, 1.00) p = 0.05	0.75 (0.57, 0.99) p = 0.04	0.67 (0.50, 0.90) p = 0.008
Emotional problems:				
Light to moderate smoking	-	0.86 (0.75, 0.98) p = 0.02	0.86 (0.76, 0.98) p = 0.03	0.86 (0.75, 0.98) p = 0.03
Heavy smoking	-	0.74 (0.64, 0.87) p<0.001	0.74 (0.63, 0.87) p<0.001	0.74 (0.63, 0.88) p<0.001
Conduct problems:				
Light to moderate smoking	-	1.47 (1.25, 1.72) p<0.001	1.46 (1.25, 1.72) p<0.001	1.44 (1.23, 1.70) p<0.001
Heavy smoking	-	1.61 (1.34, 1.94) p<0.001	1.62 (1.35, 1.96) p<0.001	1.55 (1.29, 1.88) p<0.001
Non-manual social class of origin:				
Light to moderate smoking	-	-	1.00 (0.78, 1.28) p = 1.00	1.09 (0.85, 1.40) p = 0.51
Heavy smoking	-	-	0.62 (0.46, 0.84) p = 0.002	0.79 (0.58, 1.09) p = 0.15
Childhood cognition:				
Light to moderate smoking	-	-	1.12 (0.98, 1.27) p = 0.10	1.20 (1.05, 1.38) p = 0.009
Heavy smoking	-	-	0.89 (0.76, 1.04) p = 0.14	1.13 (0.95, 1.33) p = 0.17
Advanced education:				
Light to moderate smoking	-	-	-	0.77 (0.58, 1.02) p = 0.07
Heavy smoking	-	-	-	0.38 (0.26, 0.55) p<0.001
Non-manual adult social class:				
Light to moderate smoking	-	-	-	0.78 (0.58, 1.03) p = 0.08
Heavy smoking	-	-	-	0.52 (0.38, 0.72) p<0.001

Model 1: Self organization adjusted for sex alone; Model 2: additionally adjusted for emotional and conduct problems; Model 3: additionally adjusted for social class of origin and childhood cognition; Model 4: additionally adjusted for educational attainment and adult social class.

With regard to the covariates in the fully adjusted model, adolescent emotional and conduct problems were, respectively, inversely and positively associated with smoking. Female sex was inversely associated with smoking, particularly heavy smoking. Advanced education and non-manual adult social class were inversely associated with heavy smoking, although there was no residual association between non-manual social class of origin and smoking. There was a positive association between childhood cognition and light to moderate smoking, although not with heavy smoking, which was strengthened after adjustment for education and adult social class, i.e. these latter variables were exerting suppressor effects (negative confounding).

In a fully adjusted model, logistic regression showed that the only variables associated with quitting smoking for any duration were female sex (inversely: OR = 0.70 [0.47, 0.98], p = 0.04), advanced education (positively: OR = 1.65 [1.03, 2.63], p = 0.04) and non-manual adult social class (positively: OR = 1.69 [1.14, 2.52], p = 0.01). Results were similar when current smokers and those who quit within 10 years since age 65+ were compared with those who quite more than 10 years ago, except that the effect of sex was no longer significant at the 5% level (sex: OR = 0.79 [0.57, 1.09] p = 0.15); advanced education: OR = 1.91 [1.30, 2.81], p = 0.001; non-manual adult social class: OR = 1.57 [1.10, 2.24], p = 0.01).

Table 3 shows the multinomial odds ratios for alcohol consumption across adulthood, comparing abstinence and heavy consumption against the light-to-moderate reference category. With only sex adjusted (Model 1), self-organization was inversely associated with abstinence, although only marginally so with heavy consumption. The odds ratio for no consumption was strongly attenuated after further adjustment for adolescent emotional and conduct problems (Model 2), and was no longer significant at the 5% level, although that for heavy consumption was relatively unchanged. Further adjustments for social class of origin and childhood cognition (Model 3), and for education and adult social class, made relatively little difference.

**Table 3:** Multinomial logistic regression coefficients for odds of abstention and heavy alcohol consumption across adulthood (ref. = light to moderate).

	Model 1	Model 2	Model 3	Model 4
Self-organization:				
No consumption	0.76 (0.64, 0.90) p = 0.002	0.91 (0.72, 1.15) p = 0.43	1.08 (0.84, 1.38) p = 0.54	1.14 (0.89, 1.48) p = 0.29
Heavy consumption	0.88 (0.77, 1.01) p = 0.07	0.91 (0.76, 1.10) p = 0.34	0.87 (0.71, 1.06) p = 0.16	0.86 (0.71, 1.05) p = 0.15
Female sex:				
No consumption	2.32 (1.61, 3.36) p<0.001	2.07 (1.43, 3.02) p<0.001	2.07 (1.42, 3.02) p<0.001	2.07 (1.40, 3.06) p<0.001
Heavy consumption	0.24 (0.17, 0.32) p<0.001	0.25 (0.18, 0.34) p<0.001	0.24 (0.18, 0.33) p<0.001	0.26 (0.19, 0.36) p<0.001
Emotional problems:				
No consumption	-	1.44 (1.19, 1.73) p<0.001	1.41 (1.17, 1.71) p<0.001	1.43 (1.18, 1.73) p<0.001
Heavy consumption	-	0.83 (0.71, 0.98) p = 0.03	0.84 (0.71, 0.99) p = 0.04	0.83 (0.71, 0.98) p = 0.03
Conduct problems:				
No consumption	-	1.09 (0.88, 1.36) p = 0.44	1.11 (0.89, 1.39) p = 0.34	1.09 (0.88, 1.37) p = 0.43
Heavy consumption	-	1.16 (0.97, 1.38) p = 0.10	1.17 (0.98, 1.40) p = 0.09	1.17 (0.98, 1.40) p = 0.09
Non-manual social class of origin:				
No consumption	-	-	0.79 (0.53, 1.16) p = 0.22	0.92 (0.62, 1.38) p = 0.69
Heavy consumption	-	-	0.86 (0.64, 1.16) p = 0.33	0.86 (0.64, 1.17) p = 0.35
Childhood cognition:				
No consumption	-	-	0.68 (0.56, 0.82) p<0.001	0.78 (0.63, 0.96) p = 0.02
Heavy consumption	-	-	1.23 (1.05, 1.43) p = 0.009	1.25 (1.06, 1.47) p = 0.01
Advanced education:				
No consumption	-	-	-	0.67 (0.41, 1.10) p = 0.11
Heavy consumption	-	-	-	1.28 (0.91, 1.80) p = 0.15
Non-manual adult social class:				
No consumption	-	-	-	0.56 (0.38, 0.83) p = 0.003
Heavy consumption	-	-	-	0.72 (0.52, 1.00) p = 0.05

Model 1: Self organization adjusted for sex alone; Model 2: additionally adjusted for emotional and conduct problems; Model 3: additionally adjusted for social class of origin and childhood cognition; Model 4: additionally adjusted for educational attainment and adult social class.

With regard to the covariates in the fully adjusted model, adolescent emotional problems were positively associated with abstinence and inversely associated with heavy consumption; whereas adolescent conduct problems were not associated with abstinence or heavy consumption at the 5% level. Female sex was positively associated with abstinence and inversely associated with heavy consumption. While neither social class of origin nor education were independently associated with alcohol outcomes at the 5% level, those of non-manual occupation were most likely to be light to moderate drinkers. Childhood cognition was inversely associated with abstinence and positively associated with heavy consumption.

## Discussion

In a national prospective birth cohort study we found that adolescent self-organization was inversely associated with smoking pack years in adulthood, independently of sex, adolescent emotional and conduct problems, occupational social class of origin, childhood cognition, educational attainment and attained occupational social class. On the other hand self-organization was not associated with quitting smoking, which was mainly predicted by education and adult social class. Nor was there an association of significant magnitude between self-organization and alcohol consumption across adulthood, after taking account of adolescent emotional and conduct problems.

Strengths of this study are: 1. the availability of independently rated (by teachers) prospective measures of adolescent mental health; 2. repeated measures of smoking and alcohol consumption; and 3. a comprehensive range of prospectively-obtained potential confounders and mediators, including measured cognitive ability in childhood. Against these strengths we should acknowledge some limitations. Adolescent self-organization was defined through secondary analysis by a relatively small number of items, rather than by an instrument specifically designed to capture this construct; however, the psychometric techniques used to distinguish this from adolescent emotional and conduct problems were rigorous, and the resulting construct was found to have discriminant [11] and predictive [11,18,19] validity. Second, both outcome measures were self-reported and therefore potentially vulnerable to error and bias. However, independent validation would require cotinine assay for smoking, and would be even more difficult for alcohol. On the other hand, the alcohol outcome was captured by 5-day diary, widely thought to be a gold standard for measuring common consumptive behaviours. Third, information on parental smoking and drinking was not available; adolescents with nicotine-dependent parents are at higher risk of early smoking in a dose-response manner [20]; and parental drinking is associated with more drinking in offspring [21]. These parental behaviours may therefore have been an uncontrolled source of confounding. Finally, and typically for longitudinal health-related studies, there was disproportional loss to follow-up in NSHD from those who were relatively less socially advantaged, had poorer adolescent mental health, including level of self-organization, and who had lower childhood cognitive ability. However, while this might have led to some degree of underestimation of association strengths, we have no reason to believe that this would have influenced the *pattern* of these associations.

With regard to previous population-based studies, our results for this outcome are consistent with those of Dunedin, that poor self-control was associated with initiating smoking at age 15 [3]. Locus of control, which is related to self-organization, was associated with reduced risk of smoking in the British 1970 birth cohort, although this was strongly attenuated by adjustment for education [22]. Our results for quitting are also broadly consistent with a study of 7 population-based surveys, including the 1946 cohort [23]; although these authors only focused on the role of childhood and adult socioeconomic position (SEP); the latter in particular was associated with quitting in 6 of these surveys, independent of childhood SEP. In regard to alcohol, while average alcohol intake was not assessed in Dunedin, poor self-control was associated with DSM-IV classified substance dependence in this study [3]; however, this outcome was not specific to alcohol, and also classified as positive if criteria were met for dependence on tobacco, prescription drugs or street drugs.

In addition to the main findings, we should also comment on the role of adolescent emotional and conduct problems, and childhood cognition. With regard to emotional problems, the inverse associations reported here with smoking and alcohol consumption are broadly consistent with those from the Aberdeen Children of the 1950s study [24]. This weighs against any suggestion of ‘self-medication’ in people with emotional problems, given that these problems show considerable life course continuity [25]. However, this apparent risk avoidance may only generalize to those with relatively mild symptoms. In the Christchurch Health & Development Study (CHDS), adolescents who met DSM-III-R criteria for depressive disorder in mid adolescence were more likely to smoke and drink alcohol in later adolescence [26]. With regard to adolescent conduct problems, positive associations between these and smoking were previously reported in CHDS, although not with alcohol dependence [27].

With regard to childhood cognition, it is worth noting that, although high IQ is commonly assumed to protect against self-hazardous behaviours, epidemiological evidence for this assumption is ambiguous. In our study childhood cognition was unexpectedly positively associated with light to moderate smoking after adjustment for educational attainment and adult social class. This apparently contradicts findings from the UK Scottish Mental Survey and Midspan studies [28], although the latter did not control for educational attainment, which our study suggests may have been exerting a suppressor effect. On the other hand early adult cognitive ability did not predict persistent smoking in the Swedish Conscription cohort even before covariate adjustment. [29] Regarding alcohol, childhood cognition was positively and independently associated with heavy consumption. This is consistent with results from the UK 1970 birth cohort, with higher ability predicting higher intake and a positive CAGE screen. [30] Along with the above association with smoking in NSHD, and the related finding that higher childhood cognition was associated with increased likelihood of illegal recreational drug use in the British 1958 birth cohort [31], this may suggest a stimulation-seeking explanation. On the other hand lower childhood cognitive ability predicted binge drinking in the 1958 cohort [32], and in the extreme case was inversely associated with alcohol-related hospital admissions and death in the Swedish Conscription study. [33]

What are the broader implications of this study? The topic of self-organization has received considerable attention in health psychology [34,35], although not without criticism for over-emphasis on individual control, and by implication individual will. [36] From the epidemiological perspective this correspondingly under-emphasizes the social and socio-historical context in which health-related choices are made, which may explain the differential results observed in the present study. In this context smoking, the outcome which was clearly associated with self-organization was also a behaviour that underwent significant reduction during the lives of NSHD members; cigarette smoking rates fell from 54% in 1948 to 27% in 1994 in the UK [37,38], accompanied by clear health education messages and a significant shift in corresponding social attitudes. Conversely, sales of alcohol roughly doubled in the UK over this interval. [39] Relevant health education messages were couched in vaguer terms about ‘sensible’ drinking (with arbitrary recommended limits in intake increased in 1995 by the UK Department of Health); research evidence began to filter through about possible health-protective effects of light to moderate consumption; and social attitudes towards alcohol consumption were more complex than they became towards smoking. These ambiguities may explain the lack of an association between self-organization and potentially hazardous alcohol consumption.

## Conclusions

In a prospective UK birth cohort study we found that teacher-rated adolescent self-organization was inversely associated with smoking over adulthood after controlling for a range of potential confounders and mediators, but not with heavy alcohol consumption or alcohol abstention. Our hypothesis, that self-organization is inversely associated with potentially hazardous adult health behaviours was therefore only partially confirmed, although this discrepancy may be understood in terms of differing public health messaging. Nevertheless, the inverse association with smoking is hardly trivial; as previously noted, smoking is the biggest single cause of premature death in high income countries. [4] Our results suggest that promotion of self-organization during development will have important consequences for health and longevity. Such promotion is indeed already a government/NGO goal, as exemplified, for example, by the UNESCO International Bureau of Education ‘Tools of the Mind’ strategy; [40] and the UK government document ‘The Children’s Plan'. [41] Early evaluation of the former is not encouraging [42], although a meta-analysis of 30 studies suggests that self-regulated learning training can be effective in early education. [43] Our study reiterates the importance of such strategies.
